# Network regulatory mechanism of ncRNA on the Wnt signaling pathway in osteoporosis

**DOI:** 10.1186/s13008-023-00086-7

**Published:** 2023-03-07

**Authors:** Fangyu An, Xiangrui Meng, Lingqing Yuan, Yanqiang Niu, Jie Deng, Zhaohui Li, Yongqi Liu, Ruoliu Xia, Shiqing Liu, Chunlu Yan

**Affiliations:** 1 Teaching Experiment Training Center, Gansu University of Chinese Medicine, Lanzhou, 730000 Gansu China; 2 The First Clinical Medical College, Gansu University of Chinese Medicine, Lanzhou, 730000 Gansu China; 3 School of Basic Medicine, Gansu University of Chinese Medicine, Lanzhou, 730000 Gansu China; 4 School of Traditional Chinese and Western Medicine, Gansu University of Chinese Medicine, Lanzhou, 730000 Gansu China

**Keywords:** Osteoporosis, NcRNA·Wnt, Bone metabolism, Network regulation

## Abstract

Non-coding RNA (ncRNA) is a type of non-protein-coding RNA molecule transcribed from the genome which performs broad regulation of a variety of biological functions in human cells. The Wnt signaling pathway is highly conserved in multicellular organisms, playing an important role in their growth and development. Increasing evidence suggests that ncRNA can regulate cell biological function, enhance bone metabolism, and maintain normal bone homeostasis by interacting with the Wnt pathway. Studies have also demonstrated that the association of ncRNA with the Wnt pathway may be a potential biomarker for the diagnosis, evaluation of prognosis, and treatment of osteoporosis. The interaction of ncRNA with Wnt also performs an important regulatory role in the occurrence and development of osteoporosis. Targeted therapy of the ncRNA/Wnt axis may ultimately be the preferred choice for the treatment of osteoporosis in the future. The current article reviews the mechanism of the ncRNA/Wnt axis in osteoporosis and reveals the relationship between ncRNA and Wnt, thereby exploring novel molecular targets for the treatment of osteoporosis and providing theoretical scientific guidance for its clinical treatment.

## Introduction

Osteoporosis (OP) is a systemic bone metabolic disease, characterized by a weakening of bone strength, destruction of the bone microstructure, and increased bone fragility, representing a common cause of fracture [1]. Age and estrogen deficiency are the principal causes of the induction of OP [2, 3]. Other systemic diseases, such as diabetes [4], systemic lupus erythematosus [5], chronic kidney disease [6], etc*.*, can also induce OP. As the global population has aged, the incidence of OP has increased incessantly. According to recent statistics, more than 75 million people around the world suffer from osteoporosis [7]. Because of the complex pathogenesis of the disease, no effective treatment currently exists. With the development of epigenetic research [8], many researchers have discovered that manipulation of the ncRNA/Wnt axis can enhance bone.

metabolism and regulate bone homeostasis, allowing a novel research direction for the treatment of osteoporosis.

Non-coding RNA (ncRNA) is a form of non-protein-coding RNA molecule that is transcribed from the genome, providing regulation for various biological functions in human cells. The principal molecules of ncRNA include microRNA (miRNA), long chain non coding RNA (LncRNA), and circular RNA (CircRNA). These ncRNA molecules mainly participate in cell proliferation, cell differentiation, and cell death [9]. Previous studies indicate that miRNA assists in maintaining the dynamic balance of bone homeostasis, LncRNA can promote bone development, while CircRNA can affect the proliferation of bone marrow mesenchymal stem cells and their differentiation into osteoblasts [10–12]. In addition, a number of studies have demonstrated that ncRNA also affects the occurrence and development of osteoporosis by targeting the key signal molecules in the Wnt signaling pathway [13].

As a classical signaling pathway, Wnt signaling has been found to regulate multiple biological processes such as cell proliferation, migration, invasion, and apoptosis [14]. The β-catenin expression of the Wnt signaling pathway regulates the occurrence and development of osteoporosis [15]. A number of researchers have discovered that Wnt inhibits the differentiation of bone marrow mesenchymal stem cells into adipocytes, while Wnt promotes the differentiation of bone marrow mesenchymal stem cells into osteoblasts, thus it promotes bone formation and increases bone mass. Activation of the Wnt pathway transforms osteogenesis [16], fully proving that the Wnt signaling pathway has an important regulatory role in osteoporosis.

Therefore, the occurrence and development of osteoporosis are controlled by the interaction between ncRNA and key target molecules in the Wnt signaling pathway, possibly suggesting a new method for diagnosing and treating osteoporosis. The present manuscript reviews the molecular mechanism and function of non-coding RNA associated with the Wnt pathway related to the occurrence and progression of osteoporosis, and seeks to reveal novel molecular targets for the treatment of osteoporosis, so as to provide scientific theoretical guidance for its clinical treatment.

### Overview of non-coding RNA

Non-coding RNA (ncRNA) refers to RNA molecules transcribed from the genome which do not code for protein, including miRNA, LncRNA, and CircRNA [17]. NcRNA is widely involved in development and differentiation, reproduction, apoptosis, and cellular reprogramming in biological organisms, and its expression has been found to be closely associated with human disease states [18, 19].

### miRNA

**Fig. 1:**
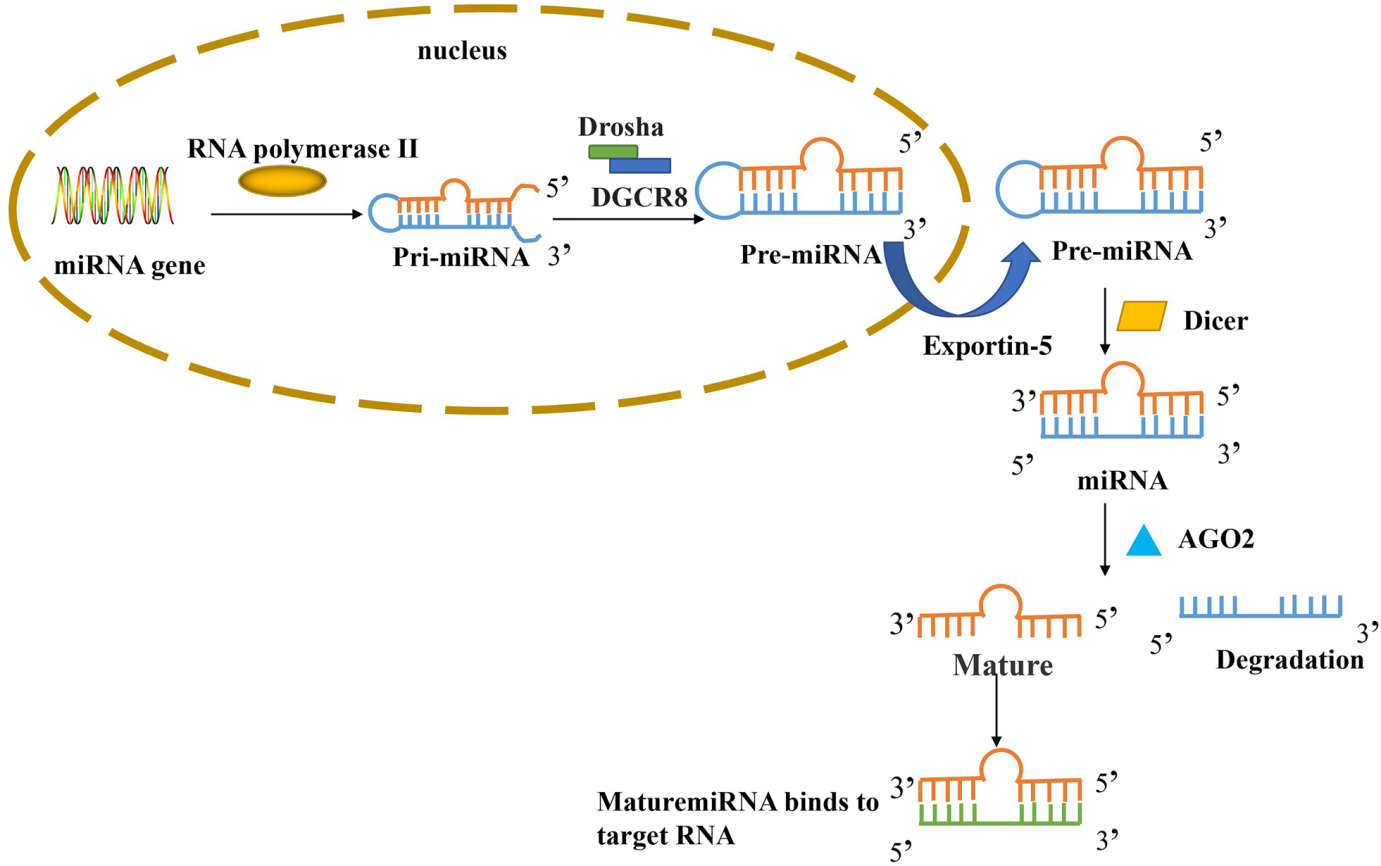
The process of biosynthesis of miRNA. The miRNA gene is transcribed into pri-miRNA under the action of RNA polymerase II in the nucleus. Pri-miRNA was cut by Drosha and DGCR8 to form pre-miRNA. Pre-miRNA is transported into the cytoplasm through the exportin-5 complex. Pre-miRNA was further cleaved by Dicer enzyme to form double-stranded miRNA. Then one miRNA chain was degraded by AGO2, and the other formed mature miRNA. Mature miRNAs bind to target mRNA

MiRNA is a class of non-coding single-stranded RNA molecules encoded by endogenous genes with a length of approximately [19–24] nucleotides [20]. They participate in the regulation of post-transcriptional gene expression in animals and plants and have a close association with multiple biological processes [21]. Many researchers have found that each miRNA molecule can have multiple target genes, while multiple miRNAs can also regulate the same target gene, and so the regulatory mechanism of each molecule or gene has a complex network relationship [22, 23]. The process of biosynthesis of miRNA is as follows (Fig. 1): The miRNA gene is transcribed into pri-miRNA under the action of RNA polymerase II in the nucleus. Pri-miRNA is cleaved by Microprocessor (a catalytic complex composed of Drosha and DiGeorge critical region 8, DGCR8) in the nucleus to form pre-miRNA, which is transported into the cytoplasm through GTP-dependent exportin-5 complex. Pre-miRNA is further cleaved by Dicer enzyme to form double-stranded miRNA, and then one miRNA chain is degraded by Argonaute 2 (AGO2), and the other one forms mature miRNA. Mature miRNAs combine with the 3'UTR of target mRNA in a complementary manner, so that the target mRNA is degraded or the translation is inhibited, so as to achieve the purpose of regulating protein expression [24–28] Plenty of studies have found that miRNA molecules participate in the occurrence and development of osteoporosis via regulation of downstream target gene expression and that of related proteins of the Wnt signaling pathway [29, 30]

### LncRNA

Long-chain non-coding RNA was first discovered in mice (Mus musculus) in 1989 [31], although no clear definition or classification of long-chain non-coding RNA existed at that time. As research on non-coding RNA increased during the twenty-first century, it was found that LncRNA is a type of non-coding RNA molecule exceeding 200 nucleotides, located in both the nucleus and cytoplasm [32]. LncRNA is similar to mRNA in structure, with a 5′ cap, 3′ polyA structure, and alternative splicing body, lacking an open reading frame and not encoding protein [33, 34] LncRNA can be divided into four categories according to its different functions: signal, bait, guide, or scaffold molecules [35]. Depending on its position relative to protein coding genes, LncRNA molecules can be classified as one of the five following categories [36]: intergene, intron, sense, antisense, or bidirectional LncRNAs. It has been found that LncRNA is involved in the pathophysiological processes associated with chronic bone diseases such as osteoporosis and osteoarthritis [37–39] Therefore, the identification of abnormal LncRNA may assist in the development of effective methods of diagnosis and treatment.

### CircRNA

CircRNA was first discovered in viruses in 1976, but due to the low abundance of transcripts, it did not attract great attention from researchers at that time [40]. As research has intensified, it has gradually been appreciated that CircRNA is a special type of non-coding RNA, forming a ring-shaped closed molecule through reverse splicing, without 5' cap or 3' polyA structures [41–45] rcRNA is characterized by functional diversity, acting not only as a sponge for miRNA, but also affecting the expression of target genes by regulating the activity of miRNA. It can also combine with active proteins to modify the biological activity of cells [46, 47]. In addition, CircRNA also acts as a transcriptional memory molecule at specific times [48–50] to record the translation of mRNA, thus representing a novel method for the identification of new molecular markers and constituting the basis for research into the analysis of the mechanism of particular diseases.

### Wnt signaling pathway in osteoporosis

In 1982, researchers identified the proto-oncogene, int1, at the integration site of a mouse breast tumor virus, and with the homology of the wingless gene of drosophila as subsequently reported by Sharma, collectively called Wnt [51, 52]. The Wnt signaling pathway is highly conserved and extremely important in multicellular organisms, playing an important role in the growth and development of organisms, in addition to other important functions [53, 54]. The Wnt signaling pathway can be categorized into two forms: the canonical Wnt/β-catenin and non-canonical Wnt signaling pathways. The non-canonical Wnt signaling pathway differs from the canonical Wnt/β-catenin pathway by including multiple β-catenin independent pathways, including the Wnt/PCP and Wnt/Ca^2+^ pathways [55, 56]. Thus, many studies have demonstrated that the Wnt signaling pathway is closely associated with the occurrence and development of osteoporosis [57–59].

### Canonical Wnt/β-catenin pathway

**Fig. 2:**
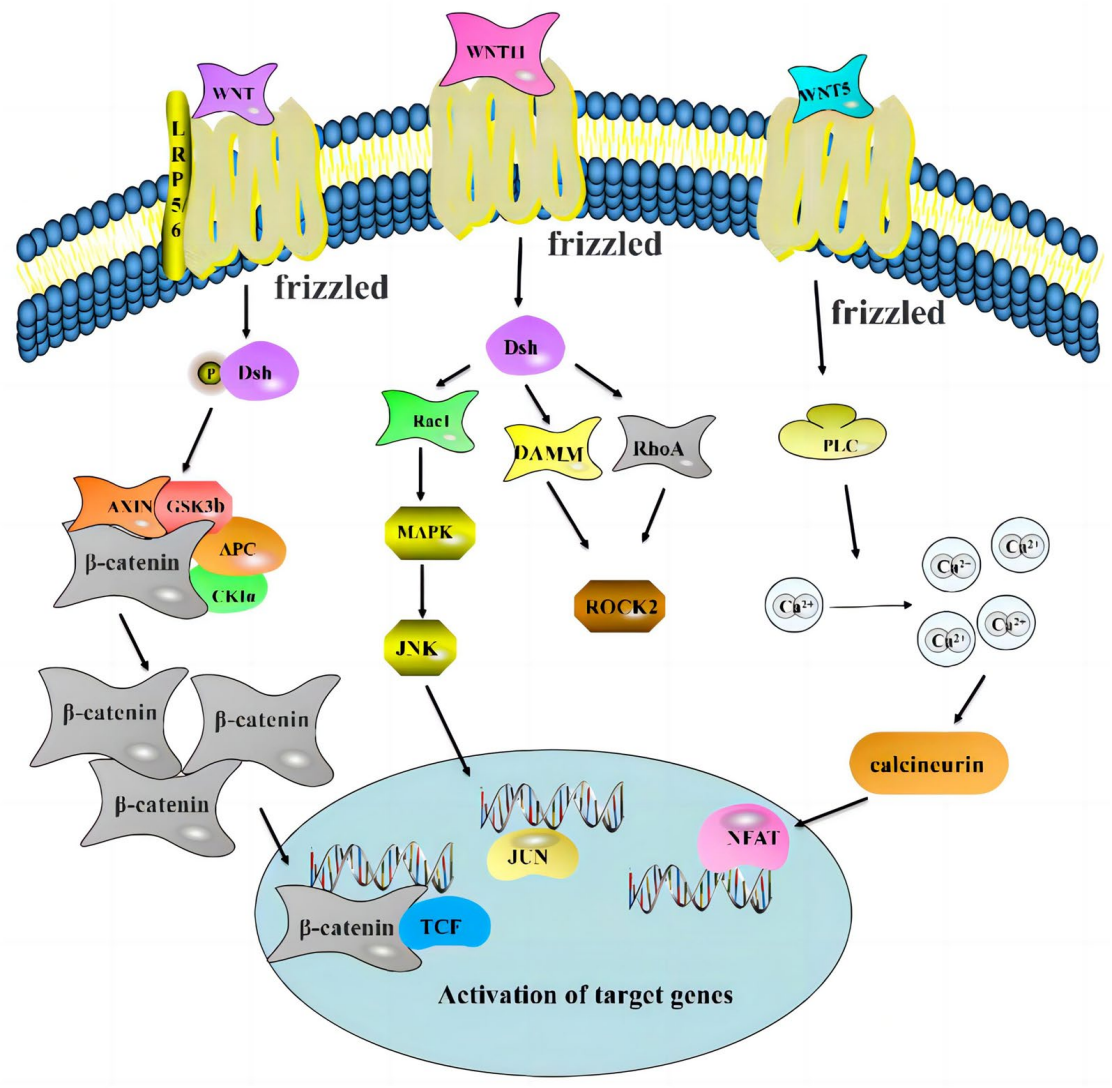
Mechanism of regulation of the Wnt signaling pathway. Wnt signaling comprises a complex regulatory network in which the Wnt protein activates downstream signaling cascades by binding to the Frizzled receptor. Wnt signaling pathways can be categorized as three different types: canonical Wnt/β-catenin, Wnt/PCP, and Wnt/Ca^2+^ pathways

The canonical Wnt/β-catenin signaling pathway is a key pathway that regulates osteoporosis. Where the Wnt ligand exists, β-catenin is not phosphorylated and it aggregates in the nucleus, thus regulating the downstream related target genes of Wnt [60]. A prominent feature of Wnt/β-catenin signaling pathway is the requirement for FZD and LRP5/6 receptors. FZD and LRP5/6 transfer Wnt signals by binding downstream cytoplasmic components to play their biological functions [61] Zheng et al*.* demonstrated that inhibition of the Wnt/β-catenin signaling pathway results in a reduction in the ability of bone marrow mesenchymal stem cells to differentiate and their ability to proliferate negatively regulated [62]. In a postmenopausal rat model of osteoporosis, Shao observed that the ubiquitin proteasome inhibitor, MG-132, delayed the occurrence and development of osteoporosis via inhibition of the degradation of β-catenin [63]. In cells cultured in vitro, Chen and other researchers discovered that Cistanoside A reduces apoptosis and activates autophagy by interaction with the Wnt/β-catenin signaling pathway, thus promoting osteogenesis of primary osteoblasts [64]. In a separate study, lycopene, a principal component of tomatoes, was shown to increase the expression levels of β-catenin, increase bone calcification, and reduce the degree of osteoporosis in patients [65]. In addition, the researchers also found that Foxf2 represses the differentiation of MSCs into osteoblasts via the Wnt pathway not only in mice but also in humans, Wnt2b is a molecular target of Foxf2 in MSCs [66]. The research described above demonstrates that that the canonical Wnt/β-catenin pathway is involved in the occurrence, development, and prognosis of osteoporosis, with a complex mechanism of action having a broad scope (Fig. 2). Further study of the complex network regulation relationships of the pathway will assist in providing theoretical support for the most beneficial treatment of osteoporosis.

#### Non-canonical Wnt/β-catenin pathway

The plane cell polarity pathway (Wnt/PCP) is one of the non-canonical Wnt signaling pathways. It mainly activates c-Jun N-terminal kinase (JNK) and causes transcription changes by activating the downstream region of Dsh, Rac, small GTPase, Rho and Cde42 [67] The non-canonical Wnt/Ca^2+^ pathway is principally activated by Wnt5a and Wnt11, which may bind to the Fzd and Ror2 receptors on the cell membrane, activate Ca^2+^-sensitive signal molecules via CaMKII and calcineurin, and cause the intracellular Ca^2+^ concentration to increase, thereby regulating cell motility and cell adhesion [56, 68–70]. As an activator of non-canonical Wnt signaling pathway, Wnt5a can jointly activate Wnt/PCP pathway and Wnt/Ca^2+^ pathway, regulate the expression of downstream factors, and play an important role in bone formation, absorption, bone loss, and matrix mineralization [71, 72]. Wan discovered that the Bj mutants have a non-synonymous point mutation in Prickle1, a core component of the non-canonical Wnt/planar cell polarity (PCP) pathway, it regulates the differentiation osteoblasts in frontal bone [73]. Li et al found that that miR-154-5p negatively regulates the osteogenic differentiation of adipose-derived mesenchymal stem cells(ADSCs) by directly targeting Wnt11 expression in the Wnt/PCP pathway under tensile stress [74]. Lin discovered that the S1PR2 antagonist JTE013 was able to promote osteogenesis via Wnt/Ca^2+^ signaling in bone marrow mesenchymal stem cells [75]. Wu et al. discovered a method of enhancing osteoporosis using a pulsed electromagnetic field to induce osteoblast formation by increasing Ca^2+^ in mesenchymal cells and activating the Wnt/Ca^2+^ signaling pathway [76]. Clarifying the mechanism of Wnt non-canonical pathway on osteoporosis can provide a new and effective treatment for prevention and treatment of osteoporosis.

To summarize, the Wnt signaling pathway is a complex protein regulatory network system, comprising principally of a group of downstream signal transduction pathways stimulated by a combination of the Wnt protein ligand and membrane protein receptors (Fig. 2). The Wnt signaling pathway performs an important role in osteoporosis. A clearer understanding of it may help in the development of novel tools for the more effective diagnosis and treatment of osteoporosis.

### ncRNA in osteoporosis

A large number of studies have confirmed that the pathogenesis of osteoporosis is associated with multiple factors such as inflammation, autophagy, oxidative stress, and an imbalance in bone homeostasis [77–79], with which ncRNA has a complex relationship.

### miRNA in osteoporosis

Autophagy is key to bone homeostasis, and its dysfunction in bone cells is related to the occurrence of osteoporosis. Lu et al*.* [80] found that the overexpression of miR-15b inhibits osteoblast differentiation and autophagy, exacerbating osteoporosis, the mechanism investigated by inhibition of the expression of USP7. Associated studies have shown that miR-151a-3p is involved in the pathogenesis of osteoporosis, promoting its progression by targeting SOCS5 and activating JAK2/STAT3 signaling. Anti-miR-151a-3p may, therefore, represent a potential therapeutic strategy for osteoporosis [81]. Li et al*.* discovered that miR-483-5p can increase the progression of osteoporosis following the analysis and comparison of the gene expression profiles of blood and bone tissue samples collected from osteoporotic patients and normal people, the mechanism of which was established as the promotion of osteoclast differentiation [82]. A number of researchers demonstrated that the high expression of miR-23a exacerbated the pathological damage from osteoporosis by inhibition of osteogenesis, promotion of bone resorption, and inflammatory polarization of macrophages, finally leading to the occurrence and further progression of osteoporosis [83]. Eric et al*.* [84] observed that miR-1270 played an important role in regulating bone metabolism. The overexpression of miR-1270 reduces the ability of human osteoblast-like cells to proliferate and migrate, thus increasing the occurrence and progression of osteoporosis. In summary, miRNA is involved in multiple processes resulting in osteoporosis. It is important that the influence of miRNA on this complex cellular process is fully revealed to improve the diagnosis and treatment of OP. Therefore, exploration of the role of different miRNA molecules in osteoporosis will be of great significance in the future.

### LncRNA in osteoporosis

Over recent years, LncRNA research in osteoporosis has become a focus of research attention. It has been found that the expression levels of the LncRNA, SNHG1, in plasma in postmenopausal osteoporosis patients are considerably lower than in the healthy population, and it has been speculated that its plasma levels could be utilized as a biomarker for the diagnosis and treatment of postmenopausal osteoporosis [85]. It has been reported that the LncRNA, PTCSC3, is upregulated in osteoporosis and the apoptosis of osteoblasts, and could, therefore, also be used as a potential therapeutic target for osteoporosis [86]. Yu et al*.* [87] found that high levels of the LncRNA, CAS11, were significantly associated with prolongation of the course of treatment and a high recurrence rate of osteoporosis in clinic. Following experiments, it was concluded that the mechanism may be related to the upregulation of TNF-α caused by the overexpression of CAS11, leading to the activation of osteoclasts and increased bone resorption. Wang et al*.* observed that the LncRNA, WT1-AS, was upregulated during osteoporosis and actively regulated the expression of p53 in osteoblasts, thereby regulating apoptosis in osteoblasts [88]. In vitro cell experiments by Qian established that knock-down of the LncRNA, AWPPH, resulted in the upregulation of type I collagen-α1 and downregulation of type I collagen-α2 expression in osteoblasts, causing the ratio of α1 to α2 to be greater than 2:1, possibly causing osteoporosis [89]. Chen found that the LncRNA, XIST, can inhibit the differentiation of osteoblasts, promote osteoporosis by targeting CUL3, and inhibit the ubiquitination and degradation of Nrf2 [90]. From the research results described above, it can be concluded that LncRNAs play a definite role in the occurrence and development of osteoporosis. There have been relatively few studies on LncRNA in osteoporosis, but the importance of the role of LncRNA cannot be ignored. A gene therapy method could be used to target LncRNAs with important therapeutic function.

### CircRNA in osteoporosis

Osteoporosis is associated with an imbalance in bone resorption compared with bone formation. It has been reported that Circ_0005564 plays a potential role in bone formation and is an active regulator of the osteogenic differentiation of bone marrow mesenchymal stem cells [91]. Zhao found that the expression of hsa_circ_0001275 increased significantly in postmenopausal osteoporosis (PMOP) patients by analysis of a CircRNA microarray, therefore representing a potential diagnostic biomarker of PMOP [92]. Xiang observed, in a separate study, that the expression of Hsa_circ_0001445 was significantly upregulated in the plasma of patients that had received anti-osteoporosis treatment, speculating that Hsa_circ_0001445 may represent a novel diagnostic biomarker for osteoporosis [93]. However, few studies have been conducted on the biological effects of CircRNA alone, most focusing on the joint effects of CircRNA and other ncRNA. However, CircRNA has potential clinical significance. In-depth studies will help to explain the molecular mechanism and biological functions of osteoporosis, and so it warrants additional study.

### Regulation of ncRNA in osteoporosis

The interaction between ncRNA molecules is currently a highly active research topic. In the currently published literature, the majority of studies have tended to investigate the regulation of downstream target gene expression using LncRNA and CircRNA via miRNA. It has been reported that in osteoporosis patients, the LncRNA, ROR, is downregulated while miR-145-5p is over-expressed. Knock down of ROR was shown to inhibit osteoblast proliferation and exacerbate the progression of osteoporosis by targeting miR-145-5p [94]. Cong demonstrated that the LncRNA, GAS5, promoted the apoptosis of osteoclasts, the mechanism of which may have been the downregulation of miR-21 expression, thus reducing the occurrence of osteoporosis [95].

Estrogen deficiency is a cause of osteoporosis in elderly women. It has been reported that estrogen can promote the expression of LncRNA H19 and regulate the osteogenic differentiation of bone marrow mesenchymal stem cells via the miR-532-3p/SIRT1 axis, thereby reducing osteoporosis [96]. A number of studies have found that the LncRNA, MSC-AS1, may upregulate BMP2 by binding miRNA-140-5p, thus promoting the osteogenic differentiation of BMSCs and preventing the progression of osteoporosis [97]. Using an hBMSC differentiation model, Li et al*.* [98] found that circ_0001795 performed the role of a molecular sponge for miR-339-5p, with Circ_0001795 downregulated in the clinical samples of OP patients, causing the occurrence and development of osteoporosis due to the ability of miR-339-5p to negatively regulate the expression of YAP1. The study demonstrated that targeting Circ_0001795 could represent a novel therapeutic target for OP. CircAtp9b enhances LPS-induced inflammation. Feng et al*.* demonstrated that up-regulation of CircAtp9b occurred after reduced formation of mature miR-17-92a, with the promotion of osteoblast apoptosis, resulting in osteoporosis [99]. Compared with normal subjects, Circ_0019693 in serum and bone tissue samples from OP patients was downregulated. It has been reported that Circ_0019693 promotes osteogenic differentiation and osteogenesis-linked angiogenesis of BMSCs by the regulation of miR-942-5p that targets PCP4, thus preventing osteoporosis [100]. The expression of Circ_0006873 was found to be upregulated in osteoporosis patients, but reduced following osteoblast differentiation. The molecular sponge miR-142-5p was found to be negatively regulated after its interaction with circ_0006873, and downregulated in osteoporosis patients while its expression increased after osteoblast differentiation. Associated experiments have demonstrated that a miR-142-5p mimic can reverse the effects of circ_0006873 overexpression on cell viability and the expression of osteogenic markers, promoting osteoblast differentiation, thus providing a new therapeutic approach for osteoporosis [101]. In summary, miRNA can function as a transport tool for LncRNA and CircRNA, thereby causing osteoporosis, so there is great potential for ncRNA to act as a research and therapeutic target for osteoporosis. At present, the studies of siRNA and piRNA about the pathogenesis of osteoporosis were still unclear. The correlational research has found that the expression of important osteogenic markers and angiogenesis related genes increased in ovariectomized mice, as the SOST siRNA was injected into ovariectomized mice by nanoparticles [102]. In another study, knock down piR-63049 could reduce bone loss in ovariectomized rats by promoting bone formation [103] Therefore, the siRNA and piRNA were used as key targeted therapy of osteoporosis and still have important research value. The existing literature demonstrates that ncRNA plays an extensive role in osteoporosis, influencing its occurrence and development and providing the potential for clinical diagnosis and treatment. However, the specific molecular biological mechanisms by which ncRNA regulates osteoporosis and the crosstalk by which these ncRNA molecules regulate osteoporosis have not yet been studied in sufficient depth, with ncRNA likely to become a research hotspot for the treatment of osteoporosis in the future. The current understanding of the role and mechanism by which ncRNA influences osteoporosis are detailed in Table 1.

**Table 1:** Role and mechanism of ncRNA in osteoporosis

	NcRNA	Effect	Effects on OP	Targeted molecule	Expression of ncRNA in OP	Refs.
MiRNA	MiR-15b	Inhibition of osteoblast differentiation and autophagy	Aggravation	USP7	↑	[80]
	MiR-151a-3p	Reduce the viability of osteoblasts	Aggravation	SOCS5, JAK2/STAT3	↑	[81]
	MiR-483-5p	Promote osteoclast differentiation	Aggravation	NFATC1, NFAT2, CTSK	↑	[82]
	MiR-23a	Inhibit osteogenesis, promote bone resorption and promote inflammatory polarization of macrophages	Aggravation	M-CSF, RANKL	↑	[83]
	MiR-1270	Reduce the proliferation and migration capability of osteoblasts	Aggravation	IRF8	↑	[84]
LncRNA	LncRNA SNHG1	–	Mitigation	–	↓	[85]
	LncRNA PTCSC3	Promote apoptosis of osteoblasts	Aggravation	–	↑	[86]
	LncRNA CAS11	Activation of osteoclasts and increased bone resorption	Aggravation	TNF-α	↑	[87]
	LncRNA WT1-AS	Promote apoptosis of osteoblasts	Aggravation	P53	↑	[88]
	LncRNA AWPPH	Collagen disorder of osteoblasts	Aggravation	–	↑	[89]
	LncRNA XIST	Inhibit osteoblast differentiation	Aggravation	CUL3, Nrf2	↑	[90]
CircRNA	Circ_0005564	Promote bone formation	Mitigation	–	↓	[91]
	Hsa_circ_000127	Promote osteoclast activation	Aggravation	β-CROSSL, OSTEOC, TP1NP	↑	[92]
	Hsa_circ_0001445	–	Mitigation	β-CTx	↓	[93]
Crosstalk regulation	LncRNA ROR/miR-145-5p	Inhibit osteoblast proliferation	Aggravation	ROR	↑	[94]
	LncRNA GAS5/miR-21	Promote osteoclast apoptosis	Mitigation	–	↓	[95]
	LncRNA H19/miR-532-3p	Regulate osteogenic differentiation	Mitigation	SIRT1	↓	[96]
	LncRNA MSC-AS1/miRNA-140-5p	Promote osteoblast differentiation	Mitigation	BMP2	↓	[97]
	Circ_0001795/miR-339-5p	Promote osteoblast differentiation	Mitigation	YAP1	↓	[98]
	CircAtp9b/miR-17-92a	Promote osteoblast apoptosis	Aggravation	–	↑	[99]
	Circ_0019693/miR-942-5p	Promote osteogenic differentiation and angiogenesis coupled with osteogenesis	Mitigation	PCP4	↓	[100]
	Circ_ 0006873/miR-142-5p	Inhibit osteoblast differentiation	Aggravation	PTEN/AKT	↑	[101]

### Role of the ncRNA/Wnt axis in osteoporosis

New evidence indicates that there are a number of clinical features of osteoporosis associated with ncRNA that are related to the Wnt pathway. In addition, the ncRNA/Wnt axis promotes the progression of osteoporosis by the regulation of multiple cellular biological functions. In this section, we will introduce the expression of the ncRNA/Wnt axis, and the corresponding clinical features, function, and mechanisms of action (Table 2).

**Table 2:** Role and mechanism of ncRNA/Wnt in osteoporosis

	NcRNA/Wnt	Effect	Effects on OP	Key molecule	Expression of ncRNA in OP	Refs.
miRNA/Wnt	miR-23b-3p/Wnt	Inhibit osteoblast differentiation	Aggravation	MRC2	↑	[104]
	miR-146a/Wnt	Inhibit osteoblast differentiation	Aggravation	DKK1	↑	[105]
	miR-210-3p/Wnt	Promote osteoblast differentiation、inhibit adipogenic differentiation	Mitigation	–	↓	[106]
	miRNA-139-5p/Wnt	Inhibition of cell proliferation、Inhibit osteoblast differentiation	Aggravation	NOTCH1	↑	[107]
	miR-26b/Wnt	Promote osteoblast differentiation	Mitigation	GSK3β	↓	[108]
	miR-200c/Wnt	Promote osteogenic differentiation and bone formation	Mitigation	Sox2、Klf4	↓	[122]
	miR-26b/Wnt	Enhanced osteogenic effect	Mitigation	–	↓	[124]
LncRNA/Wnt	LncRNA HOTAIR/Wnt	Inhibit osteoblast differentiation	Aggravation	–	↑	[109]
	LncRNA DANCR/Wnt	Inhibit osteoblast differentiation	Aggravation	–	↑	[110]
	LncRNA AK039312/Wnt	Inhibition of osteoblast differentiation and bone formation	Aggravation	miR-199b-5p、GSK-3β	↑	[111]
	LncRNA AK079370/Wnt					
	LncRNA Crnde/Wnt	Promote osteogenic differentiation	Mitigation	–	↓	[112]
	LncRNA p21/Wnt	Inhibit osteoblast differentiation	Aggravation	E2	↑	[113]
CircRNA/Wnt	circ_0024097/Wnt	Promote osteogenic differentiation	Mitigation	YAP1	↓	[114]
	Circ_ FBLN1/Wnt	Promote osteoblast differentiation	Mitigation	FZD4	↓	[115]
	circStag1/Wnt	Promote osteoblast differentiation	Mitigation	Lrp5/6	↓	[116]
Crosstalk regulation	LncRNA SNHG1/miR-181c-5p/Wnt	Inhibit osteoblast differentiation and angiogenesis,、 promote osteoclast formation	Aggravation	SFRP1	↑	[117]
	LncRNA SNHG1/miR-101/Wnt	Inhibition osteoblast differentiation	Aggravation	DKK1	↑	[118]
	LINC02381/miR-21/Wnt	Inhibition osteoblast differentiation	Aggravation	KLF12	↑	[119]
	LNC-H19/miR-141/Wnt	Promote osteoblast differentiation	Mitigation	–	↓	[123]
	LNC-H19/miR-22/Wnt					
	CircSmg5/miR-194-5p/Wnt	Promote osteoblast differentiation	Mitigation	Lrp5/6	↓	[120]
	Circ_0067680/miR-4429/Wnt	Promote osteoblast differentiation	Mitigation	CTNNB1	↓	[121]

### Role of the miRNA/WNT axis in osteoporosis

**Fig. 3:**
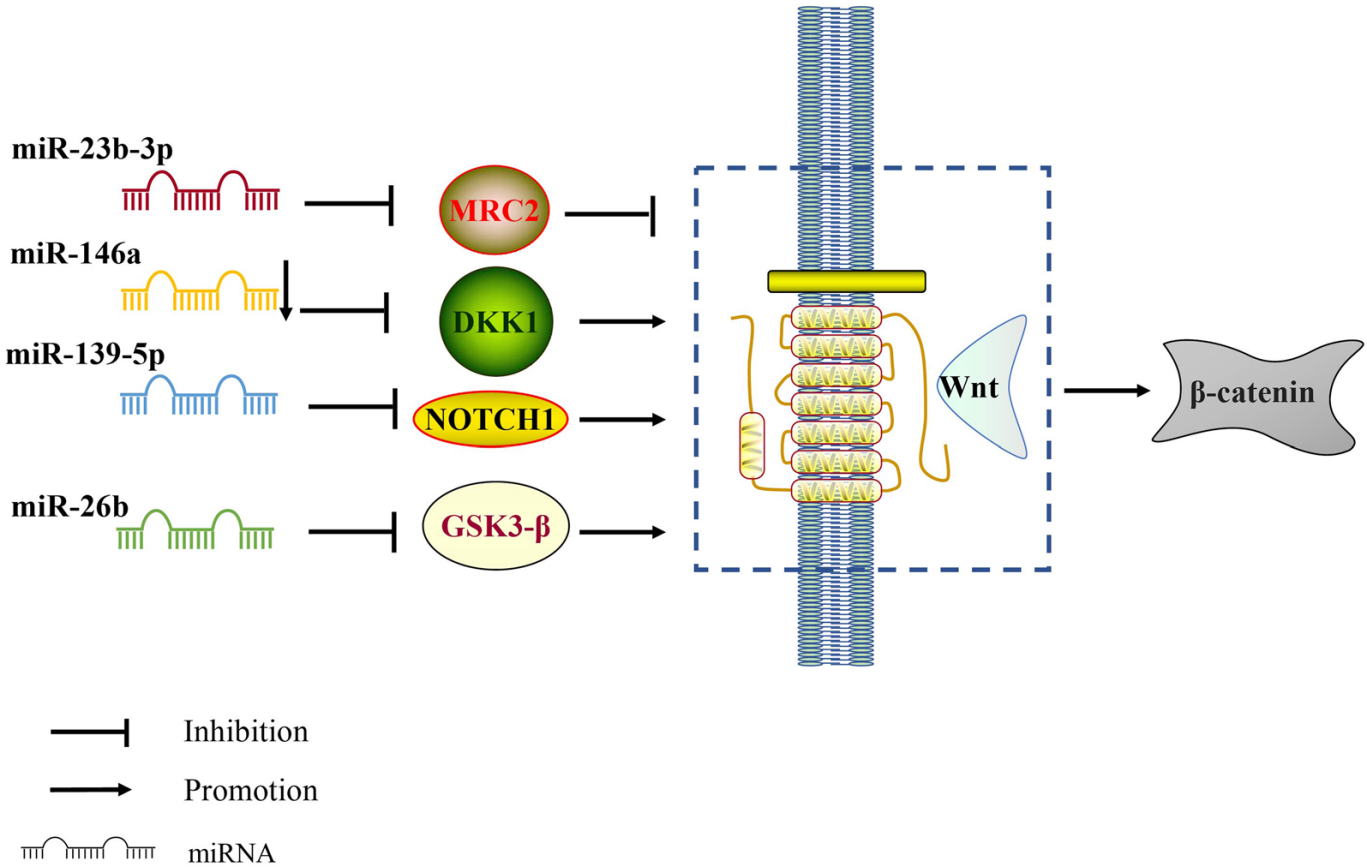
Regulation of miRNA/Wnt axis in osteoporosis. miR-23b-3p targets MRC2 which inhibits the Wnt/β-catenin pathway; miR-146a inhibits the expression of DDK1 and activates the Wnt/β-catenin signaling pathway; Downregulation of miR-139-5p inhibits Wnt/ β-catenin and induces NOTCH1 signaling in BMSCs; miR-26b directly targets GSK3β, thereby activating canonical Wnt signaling

MiRNA and Wnt signaling pathways play key roles in the development of osteoporosis. A study by Li indicated that silencing miR-23b-3p can reduce osteoporosis in mice. In addition, knock-down of miR-23b-3p promoted the osteogenic differentiation of hMSCs by upregulation of the expression of Runx2, OCN, and Osterix, thereby enhancing the activity of ALP. Mechanistically speaking, MRC2 is the downstream target gene of miR-23b-3p, a miRNA that inhibits the Wnt/β-catenin signaling pathway during the process of hMSCs osteogenic differentiation by targeting MRC2, thus promoting their osteogenic differentiation and delaying the manifestation of osteoporosis, providing a novel perspective on a potential treatment for osteoporosis [104]. Liu et al*.* discovered that downregulation of miR-146a promoted osteogenic differentiation and inhibited the expression of DDK1, which was observed following activation of the Wnt/β-catenin signaling pathway. Therefore, downregulation of miR-146a was able to relieve osteoporosis and so represents a potential therapeutic target for osteoporosis [105]. It has been reported that in ERα-deficient rat bone marrow mesenchymal stem cells, the overexpression of miR-210-3p promotes osteogenic differentiation and inhibits adipogenic differentiation, possibly associated with the Wnt signaling pathway, providing new insight into the mechanism of bone metabolism regulation [106]. In an osteoporotic rat model, the expression of miRNA-139-5p increased compared with the normal group, while the downregulation of miRNA-139-5p promoted the osteogenic differentiation of BMSCs by targeting the Wnt/β-catenin signaling pathway with NOTCH1 [107]. Other researchers have also found that overexpression of miR-26b resulted in the key regulatory factor, GSK3β, in the Wnt signaling pathway to be downregulated, resulting in β-catenin activation. MiR-26b promotes BMSC osteogenesis by directly targeting GSK3β and activating the canonical Wnt signaling pathway, indicating that it may be a potential therapeutic target for osteoporosis [108]. In summary, the biological role of the miRNA/WNT axis in osteoporosis is mostly associated with osteogenesis, modulating the manifestation and progression of osteoporosis via the promotion or inhibition of osteogenesis-related gene expression (Fig. 3). Exploration of effective target molecules of the miRNA/Wnt axis during the development of osteoporosis will provide a broader basis for its targeted molecular therapy.

### Role of LncRNA/Wnt axis in osteoporosis

**Fig. 4:**
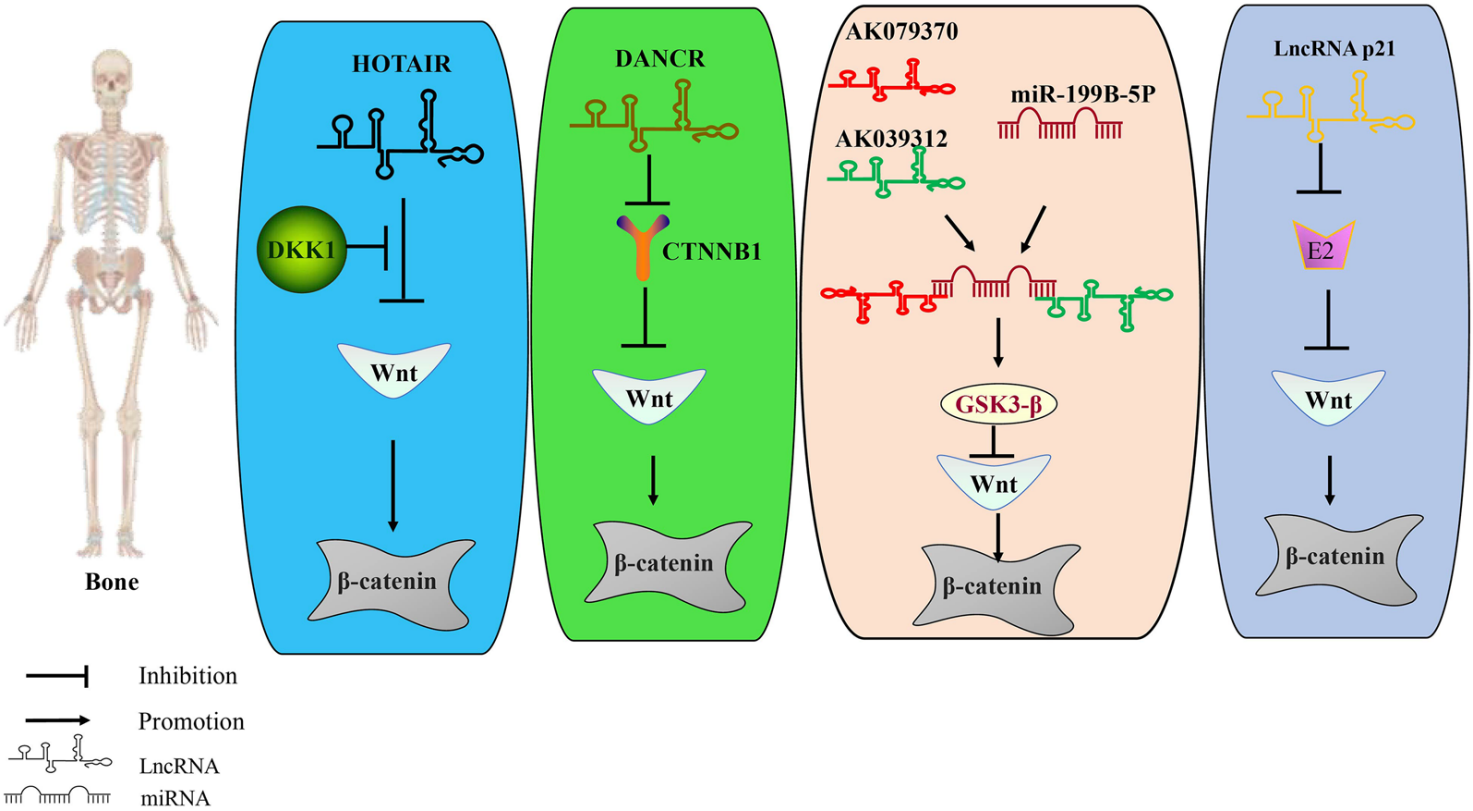
Regulatory mechanism of the LncRNA/Wnt axis in osteoporosis. The LncRNA, HOTAIR, inhibits the Wnt signaling pathway, with DKK1 able to inhibit this process; The LncRNA, DANCR, regulates the Wnt/β-catenin signaling pathway by inhibition of CTNNB1; The LncRNAs, AK039312 and AK079370, bind and chelate miR-199b-5p, thereby inhibiting the Wnt/β-catenin pathway

With the wide use of gene chips, genomics, and other high-throughput gene sequencing analytic technologies, increasing numbers of LncRNA molecules have been identified that play both positive and negative roles in the regulation of the occurrence and progression of osteoporosis. Shen demonstrated that the LncRNA, HOTAIR, inhibits the osteogenic differentiation of BMSCs by regulating the expression of the osteogenic gene, RUNX2D, via downregulation of the Wnt/β-catenin pathway [109]. Wang demonstrated that the LncRNA, DANCR, and miR-320a regulate the Wnt/β-catenin signaling pathway via inhibition of CTNNB1, inhibiting osteogenic differentiation. Co-expression of miR-320a and DANCR resulted in additive inhibitory effects. These observations highlight the potential for DANCR and miR-320a to act as therapeutic targets for osteoporosis [110]. Yin observed that the LncRNAs, AK039312 and AK079370, could both inhibit osteoblast differentiation and bone formation by the downregulation of osteoblast transcription factor expression. This inhibition was achieved by binding and chelating miR-199b-5p, enhancing GSK-3β expression that further inhibited the Wnt/β-catenin pathway [111]. Researchers have found that the proliferation and differentiation of osteoblasts were considerably inhibited in mice following the knockout of the gene for the LncRNA, Crnde, a bone metabolism modulator, causing the development of a low bone mass phenotype [112]. Additional research by Mieradili indicated that Crnde regulates bone formation through Wnt/β-catenin signal transduction [112]. Yang also confirmed that reduced expression of the LncRNA, p21, activates the Wnt/β-catenin signaling pathway by increasing E2 secretion, finally stimulating bone formation and the increased osteogenic differentiation of mesenchymal stem cells in OP model rats, playing a role in regulating the balance of bone metabolism [113]. In the studies above, the LncRNA/Wnt axis also played an important regulatory role in the occurrence and development of osteoporosis (Fig. 4), influencing the expression of associated proteins and genes via the regulation of cytokines, thus controlling the pathophysiological process of osteoporosis.

### Role of the CircRNA/Wnt axis in osteoporosis

**Fig. 5:**
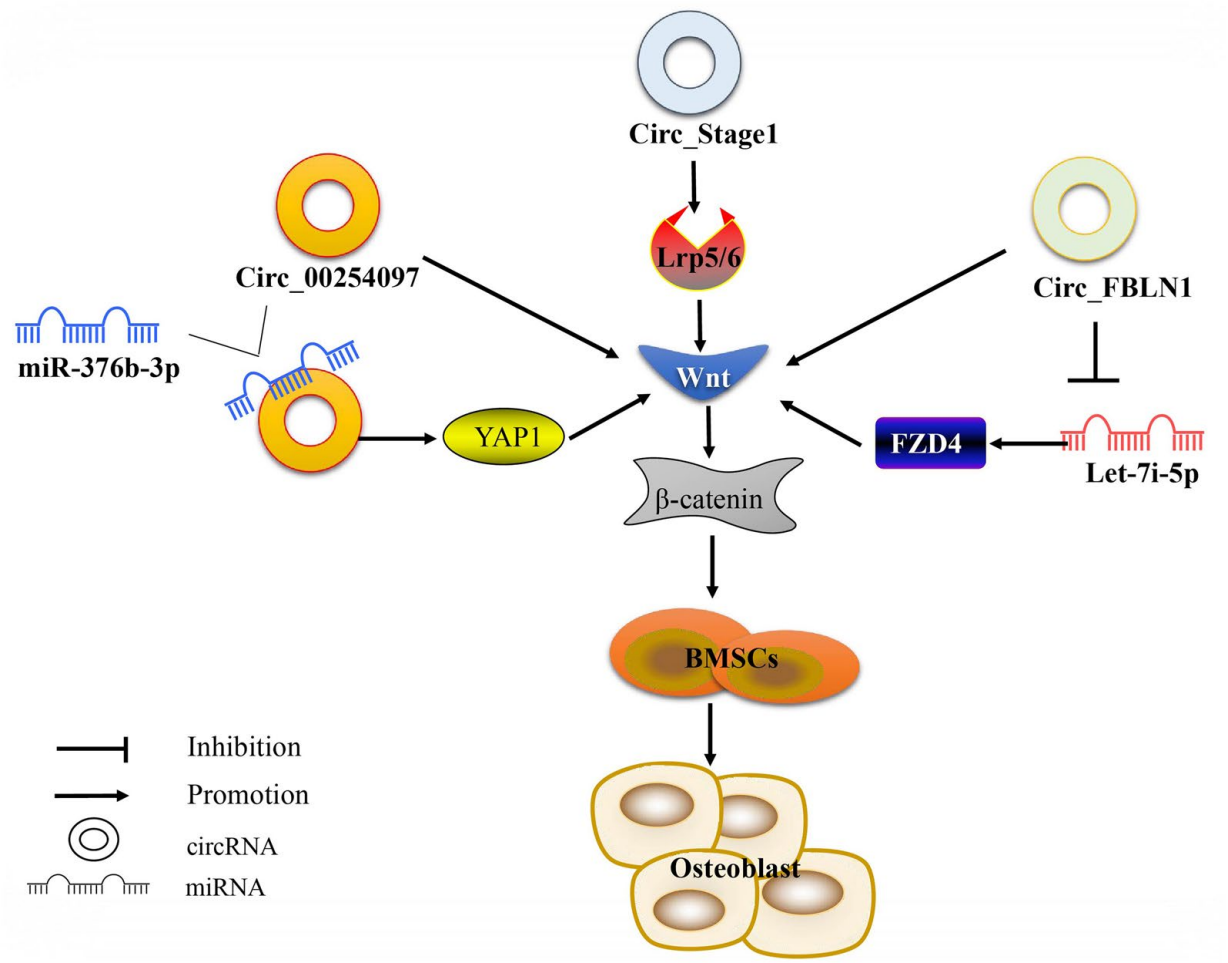
Regulatory mechanism of the CircRNA/WNT axis in osteoporosis. circ_0024097 promotes osteoblastic differentiation via the miR-376b-3p/YAP1 axis and Wnt/β-catenin pathway; circ_FBLN1 promotes the proliferation and osteogenesis of hBMSCs by the regulation of the let-7i-5p/FZD4 axis and Wnt/β-catenin pathway; CircStag1 activates the Wnt signaling pathway by stabilizing Lrp5/6 and β-catenin mRNA, thereby promoting the osteogenesis of BMSCs

Compared with the miRNA/Wnt and LncRNA/Wnt axes, the role of the CircRNA/Wnt axis in osteoporosis has been studied to a lesser degree, although its role should not be underestimated. Huang observed for the first time that circ_0024097 promoted osteogenic differentiation via the miR-376b-3p/YAP1 axis and Wnt/β-catenin pathway, thus reducing osteoporosis [114]. Silencing of Circ_FBLN1 inhibited the proliferation and osteogenic differentiation of hBMSCs, with analysis of the mechanism indicating that the process occurred by regulation of the Let-7i-5p/FZD4 axis and inhibition of the Wnt/β-catenin pathway [115]. CircStag1 is an active regulator of osteogenesis and bone regeneration and it has been shown that overexpression of CircStag1 in vivo promotes bone formation [116]. Chen found that CircStag1 activates the Wnt signaling pathway by stabilization of Lrp5/6 and β-catenin mRNA, thus promoting the osteogenesis of BMSCs, indicating that CircStag1 is a potential therapeutic target for osteoporosis [116]. Current research on the CircRNA/Wnt axis remains at a preliminary, exploratory stage, as the vast majority of CircRNA molecules has not yet been studied, and so their functions remain unknown (Fig. 5). It is anticipated that the mechanisms of the CircRNA/Wnt axis will be a focus of research interest in the future, providing a new theoretical basis for the treatment of osteoporosis.

### Role of the ncRNA in Wnt signal pathway

**Fig. 6:**
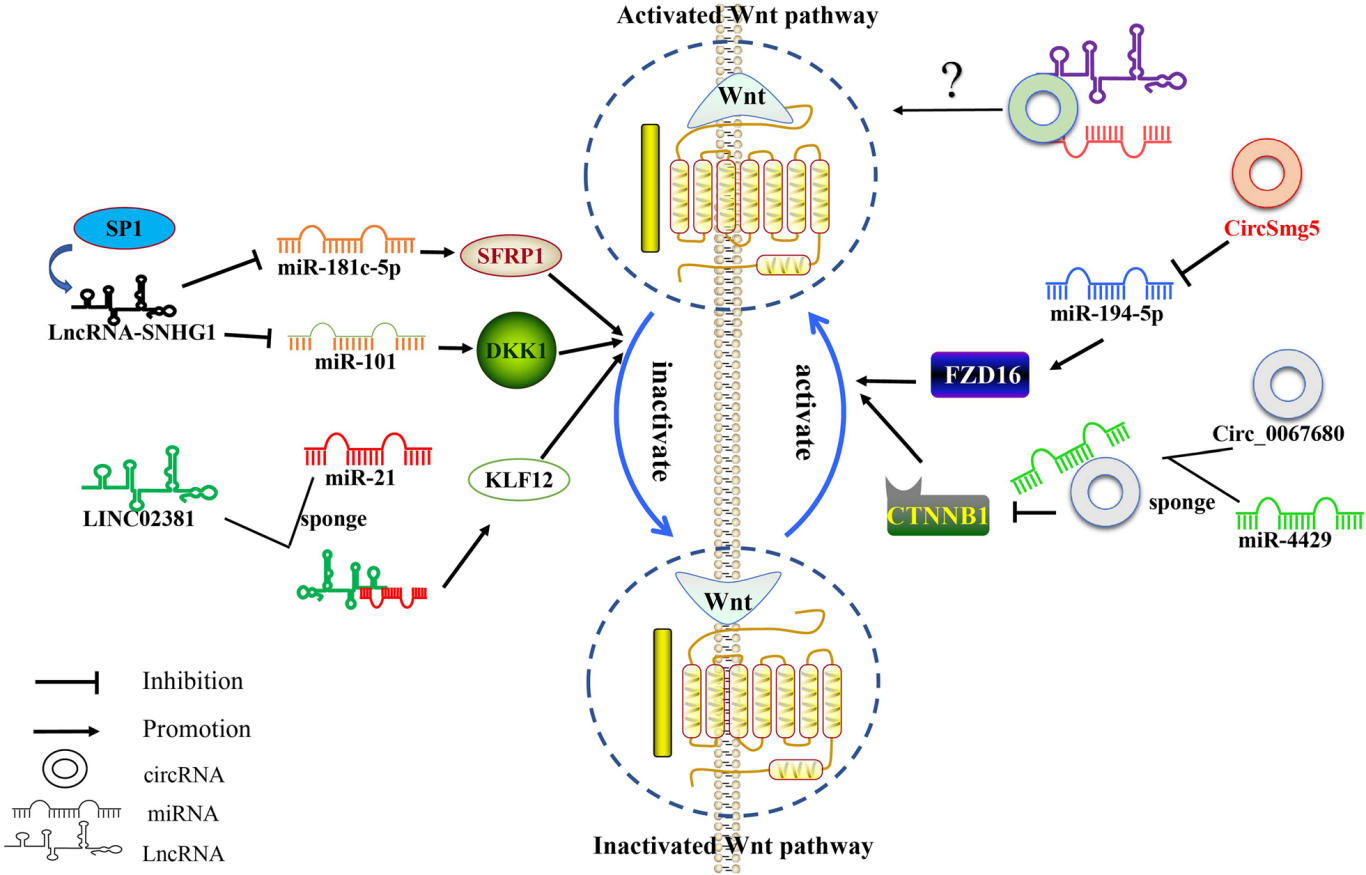
Mechanism of ncRNA in osteoporosis via the Wnt signaling pathway. miR-181c-5p acts as a sponge-regulated Wnt/β-catenin signaling pathway for the LncRNA, SNHG1; LINC02381 acts as a sponge for miR-21. LINC02381 inhibits the Wnt/β-catenin signaling pathway by absorbing miR-21; CircSmg5 activates the Wnt/β-catenin signaling pathway by targeting the miR-194-5p/Fzd6 axis; Circ_0067680 activates the Wnt/β-catenin signaling pathway by sequestering miR-4429 as a ceRNA, thereby regulating the expression of CTNNB1; The co-regulation mechanism of miRNA by LncRNA and CirRNA remains unknown

MiRNA, CircRNA and LncRNA have complex network regulatory relationships. According to current research, the three types of ncRNA not only independently regulate the Wnt signaling pathway to act on osteoporosis, but also regulate crosstalk and together play a biological role (Fig. 6). Studies have shown that the LncRNA, SNHG1, after induction by SP1, can regulate the SFRP1/Wnt/β-catenin signaling pathway via the sponge, miR-181c-5p, thus inhibiting osteoblast differentiation and angiogenesis, and promoting osteoclast formation. Silencing of SNHG1d may provide a potential treatment for osteoporosis [117]. Xiang et al*.* observed that the expression of SNHG1 was downregulated in a time-dependent manner during osteogenic differentiation, while its overexpression was shown to inhibit osteogenic differentiation. Additional studies have confirmed that SNHG1 regulates the Wnt/β-catenin signaling pathway and reduces BMSC osteogenic differentiation via the miR-101/DKK1 axis as competitive endogenous RNA [118]. Zhao concluded that LINC02381 acts as a sponge for miR-21, and inhibits the osteogenic differentiation of hUS-MSCs by absorbing miR-21, thereby enhancing the inactivation of the Wnt/β-catenin pathway mediated by KLF12 [119]. It has been reported that CircSmg5 is downregulated in a mouse model of osteoporosis, although the mechanism is unknown [120]. Yue has concluded that it promotes BMSC osteogenic differentiation by targeting the miR-194-5p/Fzd6 axis, activating Wnt/β-catenin signal transduction [120]. Circ_0067680 is derived from the differential protein kinase domain 2A (C3orf58) of the host gene and is upregulated during the osteogenic differentiation of hBMSCs. Huang demonstrated that circ_0067680 regulates the expression of catenin β1 (CTNNB1) by sequestering miR-4429 as a competing endogenous RNA (ceRNA) molecule, thus activating the Wnt/β-catenin signaling pathway, suggesting that it may represent a potential biomarker of osteoblast differentiation [121]. In conclusion, miRNA, CircRNA, and LncRNA constitute a complex gene regulatory pathway, interacting and influencing each other to form a rich gene regulatory network. At present, the research on this complex regulation mechanism in osteoporosis is lacking. The majority of studies only summarize the influence of LncRNA and CirRNA on miRNA and have not explored the common regulatory mechanisms of LncRNA and CirRNA on miRNA. With the continuous development of technology in molecular biology, the mechanisms of modification and regulation of the ncRNA/Wnt axis of osteoporosis target genes become continuously revealed, allowing more accurate screening, diagnosis and leading to better treatment of osteoporosis in the future.

### Role of ncRNA/Wnt pathways on bone remodeling

Bone remodeling is the core of osteoporosis, and the maintenance of bone mass mainly depends on the balance between osteoblastic bone formation and osteoclast bone absorption. As an important physiological process, bone remodeling is regulated by a variety of cytokines. Adil and other scholars have found that miR-200c can promote osteoblast differentiation and bone regeneration by targeting Sox2 and Klf4 to activate Wnt signals, which can be used as an effective gene therapy tool for human clinical bone reconstruction [122]. Liang et al. proved through experiments that the stable expression of LNC-H19 can significantly accelerate the differentiation of osteoblasts in vivo and in vitro, and its mechanism may be achieved by competitively inhibiting the functions of miR-141 and miR-22 to activate WNT signals [123]. As one of the important cytokines, miR-26b can be activated directly β-catenin enhances the osteogenic effect and plays an important role in bone reconstruction [124]. From the above research, we found that the ncRNA/WNT axis plays an important regulatory role in the process of bone reconstruction, and maintaining the dynamic balance process of bone reconstruction helps to further find new targets for the treatment of osteoporosis, which will provide more effective and convenient means for clinical work.

## Conclusion and prospects

In the past, ncRNA has been considered a transcriptional by-product with no particular function and without direct participation in protein coding. However, as epigenetic research has become more sophisticated, ncRNA has been found to be important in the manifestation and development of osteoporosis. Additionally, the Wnt signaling pathway is also involved in cell proliferation, differentiation, apoptosis, and oxidative stress and inflammatory reactions. Recent evidence suggests that the ncRNA/Wnt axis regulates the expression of genes related to bone metabolism and influences the progression of osteoporosis. We have found that ncRNA is the key therapeutic target for OP, and the majority ncRNA modifies osteoblast-related genes via the canonical Wnt/β-catenin pathway, resulting in an imbalance in bone homeostasis that induces osteoporosis. Over recent years, a series of ncRNA/Wnt axis inhibitors have been identified. Catenin-β-interacting protein 1 (CTNNBIP1) is an inhibitor of Wnt/β-catenin signaling, although its role in osteogenesis remains unclear. Zhang et al*.* demonstrated that miR-486-3p targets CTNNBIP1, thus activating the Wnt/β-catenin signaling pathway and promoting BMSCs to undergo osteogenic differentiation [125]. ncRNA is now regarded as a novel biomarker for osteoporosis, gradually being used in the early diagnosis of osteoporosis. However, the majority of studies of the ncRNA/Wnt axis have utilized tissues and cells in vitro, and the results should be verified by additional animal experiments and clinical trials, to provide a more clear theoretical basis for the clinical treatment of osteoporosis.

As the global population ages, osteoporosis has become one of the most common and costly diseases around the world. Osteoporosis patients are prone to fracture, and severe cases may even lead to death. In addition, osteoporosis has a considerable effect on the health and quality of life of patients, causing a heavy socio-economic burden with major social problems. The manifestation and development of osteoporosis are regulated by multiple complex factors, and so there is a need to innovate with novel treatment methods and the development of more effective drugs to prevent and treat osteoporosis. By summarizing the mechanisms of the ncRNA/Wnt axis in osteoporosis, we have shown that ncRNA plays an important role in regulating bone homeostasis, and represents a basis for screening new therapeutic targets for osteoporosis, in addition to using it as a key target for new drug research and development.

We have also found that the research on ncRNA in osteoporosis is mostly limited to the canonical Wnt/β-catenin signaling pathway and mechanisms of osteoblast metabolic regulation, while the non-canonical Wnt signaling pathway and the mechanisms of the metabolic regulation of osteoclasts and other forms of metabolic regulation are somewhat limited. Therefore, the study of ncRNA on nonclassical Wnt signaling pathways, and mechanisms of osteoclast metabolic regulation and other metabolic regulation in osteoporosis should be more prominent in the future.

## Data Availability

Not applicable.

## Abbreviations

ncRNA: Non-coding RNA
OP: Osteoporosis
LncRNA: Long chain non coding RNA
CircRNA: Circular RNA
UTR: 3′-Untranslated region
SOCS5: Signal transducer and activator of transcription 5 antibody
JAK2/STAT3: Nonreceptor tyrosine protein kinase 2/signal transducer and activator of transcription 3
BMSCs: Bone marrow mesenchymal stem cells
hMSCs: Human bone marrow mesenchymal stem cells
OCN: Osteocalcin
AL: P.alkaline phosphatase
MRC2: Mannose receptor C type 2
DDK1: Dickkopf related protein 1
GSK-3β: Glycogen synthase kinase-3β
Lrp5/6: Low density lipoprotein receptor related protein 5/6
CTNNBIP1: Catenin-β-interacting protein 1
